# Endovascular Stent-Graft Repair of the Ascending Aorta: Assessment of a Specific Novel Stent-Graft Design in Phantom, Cadaveric, and Clinical Application

**DOI:** 10.1007/s00270-021-02859-5

**Published:** 2021-06-27

**Authors:** Sven R. Hauck, Alexander Kupferthaler, Marlies Stelzmüller, Wolf Eilenberg, Marek Ehrlich, Christoph Neumayer, Florian Wolf, Christian Loewe, Martin A. Funovics

**Affiliations:** 1 Cardiovascular and Interventional Radiology, Department of Bioimaging and Image-Guided Therapy, Medical University of Vienna, Vienna, Austria; 2 Department of Cardiothoracic Surgery, Medical University of Vienna, Vienna, Austria; 3 Department of Vascular Surgery, Medical University of Vienna, Vienna, Austria; 4 Department of Diagnostic and Interventional Radiology, Ordensklinikum Linz, Linz, Austria

**Keywords:** Ascending aortic stent-graft, Custom-made stentgraft, Ascending TEVAR

## Abstract

**Purpose:**

To test a stent-graft specifically designed for the ascending aorta in phantom, cadaver, and clinical application, and to measure deployment accuracy to overcome limitations of existing devices.

**Methods:**

A stent-graft has been designed with support wires to fixate the apices toward the inner curvature, thereby eliminating the forward movement of the proximal end which can happen with circumferential tip capture systems. The device was deployed in three aortic phantoms, and in four cadavers, deployment precision was measured. Subsequently, the device was implanted in a patient to exclude a pseudoaneurysm originating from the distal anastomosis after ascending aortic replacement.

**Results:**

The stent-grafts were successfully deployed in all phantoms and cadavers. Deployment accuracy of the proximal end of the stent-graft was within 1 mm proximally and 14 mm distally to the intended landing zone on the inner curvature, and 2–8 mm distal to the intended landing zone on the outer curvature. In clinical application, the pseudoaneurysm could be successfully excluded without complications.

**Conclusion:**

The novel stent-graft design promises accurate placement in the ascending aorta. The differential deployment of the apices at the inner and outer curvatures allows deployment perpendicular to the aortic axis.

**Level of Evidence:**

No level of evidence.

## Introduction

While endovascular repair of the descending aorta using a stent-graft is a well-established procedure [1–5], fewer reports exist on the application of this technique in the ascending aorta [6–31]. Commercially available stent-graft devices designed for use in the descending thoracic or abdominal aorta [32] are of limited use mainly due to length and size restrictions as well as lack of control during deployment [10, 12, 33]. Recently, custom-made devices have been successfully implanted [26] and some manufacturers are beginning to offer devices for ascending TEVAR (aTEVAR), such as the Zenith Ascend (Cook Medical, Bloomington, IN), Nexus (Jotec, Hechingen, Germany), and the TAG Arise (Gore, Flagstaff, AZ) which is evaluated in a feasibility study [34]. One major limitation is control during deployment in a curved aortic arch via femoral access. The tips of the most proximal stent segment (apices) are in most devices clasped to the central cannula to prevent distal migration during deployment before firm contact with the aortic wall is made. While this technique provides adequate control in a straight aortic segment, deployment becomes erratic in the curve of the aortic arch. Both, proximally clasped devices and devices with free apices at the inner curvature have their inherent limitations [35].

In this study, a device, which has been designed specifically for use in the ascending aorta from a femoral access, uses differential control systems for the apices at the outer and inner curvatures. The prototype was deployed in aortic phantoms and in cadavers. The primary objective of these experiments was to determine the deployment accuracy by measuring the distance between the pre-specified and the actual landing zone. Subsequently, the stent-graft was deployed in a patient with an ascending aortic pseudoaneurysm diagnosed four weeks after replacement surgery for type A dissection.

## Methods

All studies were undertaken in compliance with legal and institutional requirements including consent from the patient and body donors.

### Stent-graft design

The novel stent-graft is based on the Relay thoracic stent-graft (Terumo Aortic, Sunrise, FL, USA) which has been described in detail elsewhere [32]. This device specifically designed for the ascending aorta is available in diameters up to 46 mm, and in lengths from 65 mm. Depending on diameter, the introducer measures 24–26 F. Graft material is densely woven polyester covering both ends supported by two to four stent segments made of 250 µm nitinol wire. The inner nitinol cannula of the deployment system is pre-curved, thereby facilitating the correct rotational position of the stent-graft.

**Fig. 1:**
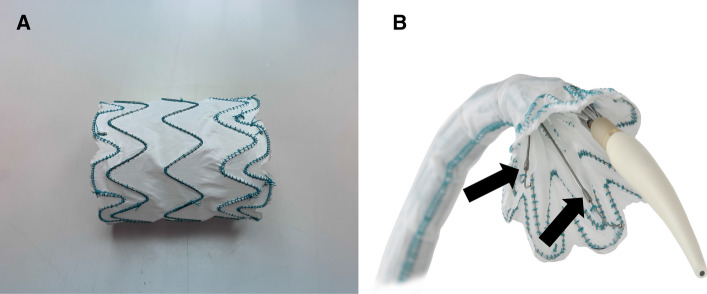
The dedicated ascending aortic stent-graft in fully (A) and partially (B) deployed state. The support wires for the apices facing the inner curvature are shown in B (black arrows). Images courtesy of Terumo Aortic

**Fig. 2:**
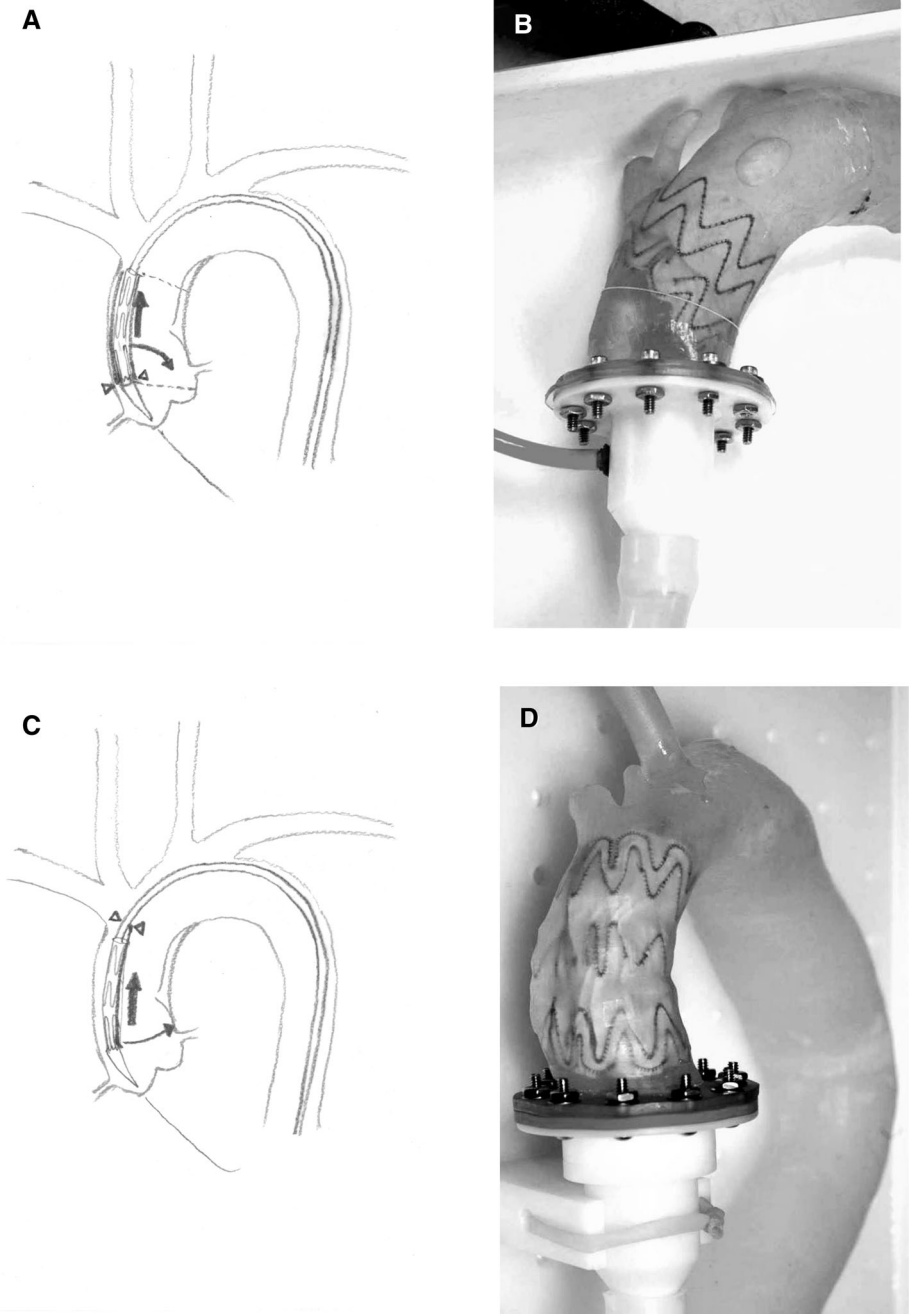
During ascending aortic stenting from a femoral approach, the stentgraft is closely appositioned to the outer curvature of the aorta. In conventional stent-grafts (A, B), the proximal apices are clasped to a proximal pivotal point at the base of the nosecone (arrowheads). During deployment, when the sheath is retracted (straight arrow), the parts of the stentgraft facing the inner curvature must move in a quarter-circle toward the inner aortic wall, since they are still attached to the nosecone (curved arrow). This creates an oblique position of the stentgraft with the inner parts dangerously close to the origin of the left coronay artery (A, dotted line, B-phantom experiment). The novel design (C, D) supports two apices facing the inner curvature with long levers from distal (arrowheads), instead of clasping them from proximal. During retraction of the sheath (straight arrow), the inner parts of the stentgraft are guided in a slightly curved path (curved arrow) toward the inner wall. This results in a predictable positioning of the proximal end of the stentgraft almost exactly perpendicular to the longitudinal axis of the ascending aorta (D-phantom experiment).

The proximal stent segment has four to six apices. The two apices facing the outer curvature are connected to the tip capture system and are only released after the stent-graft is fully deployed. The particular novelty is that the two apices facing the inner curvature are not connected to the tip capture system but are braced by two levers (support wires) of 45 mm in length, which originate from the central cannula inside the stent-graft (Fig. 1). These levers ensure that the part of the proximal end of the stent-graft facing the inner curvature is not pulled forward during deployment but controlled from a more distal point of the stent-graft. As a result, the proximal end of the stent-graft can be positioned exactly perpendicular to the aortic axis (Fig. 2). The system is advanced in its rigid sheath to the descending aorta. While the first prototypes (used in the cadaver tests) had a longer nosecone such as the TEVAR stentgrafts, the recent prototypes (used clinically) have a shortened nosecone of 22 mm length. This avoids the need to pass a guidewire through the aortic valve if the proximal landing zone is at sufficient distance from the valve. From this point, the device is advanced in a soft nylon sheath of 12 mm in diameter to its final deployment position. When the nylon sheath is retracted, the stent-graft is fully expanded, with the two outer apices still affixed to the nose cone, and the two inner apices held by the two levers, but already in contact to the aortic wall at the inner curvature. In the final step, all apices are released and the delivery system is retracted.

### Phantom Studies

An aortic model with a Type III aortic arch [36] and an ascending aortic diameter of 48 mm was created from a 3D CT dataset from resin using stereolithography and perfused from the valve to the right common femoral artery using pump and reservoir with constant temperature of 37 °C and a flow of 4 l/min.

A circular marker was placed around the ascending aorta 1 cm distally to the origin of the left coronary artery and perpendicular to the central axis of the aorta at this level. The stent-graft was then deployed via a femoral access as close as possible to the marker. After deployment, the longitudinal distance between the marker and the proximal end of the stent-graft was measured at the outer and the inner curvatures of the aorta with a caliper. Three deployments were executed in the same model.

### Cadaver Studies

Four preserved human cadavers were placed in prone position on a radiolucent table. The cadavers were not perfused and had ascending aortic diameters below 40 mm. A metal marker was placed at the outer wall of the ascending aorta, 1 cm distally to the origin of the left coronary artery.

Under fluoroscopic guidance and injection of diluted contrast medium, the stent-graft was inserted and deployed with its proximal end as closely as possible to the marker. The distance between the proximal end of the stent-graft and the marker was measured on the fluoroscopic images.

### Clinical Application

A 76-year-old man had undergone open surgical ascending aortic replacement with a 32-mm surgical graft and suffered from a pseudoaneurysm of 12 × 18 × 9 mm at the inner curvature with its entry at the level of the distal anastomosis. Nine weeks after the operation, repeat CT showed the pseudoaneurysm had increased to 29 × 38 × 15 mm and was partially compressing surrounding structures.

A second operation was deemed too high risk for the patient because of advanced age, previous myocardial infarction, and generalized atherosclerosis. Since the distances between the entry tear of the pseudoaneurysm and the left coronary artery, as well as between the entry tear and the brachiocephalic trunk were >20 mm along the aortic axis, a multidisciplinary team evaluation favored endovascular repair.

Ascending TEVAR was undertaken 12 weeks after the index procedure. The origins of the left coronary artery and the brachiocephalic trunk were identified. Visualization of the entry of the pseudoaneurysm was complicated by slow flow and extensive overlay of the lumina. Due to the length of the entry, an ascending aortic length of 85 mm, and the lack of stent-grafts longer than 65 mm at the time, two 42 × 65 mm stent-grafts were implanted via a femoral access.

### Data Evaluation

The position of the proximal end of the stent-graft relative to the intended landing zone was recorded in all deployments. Data were presented as boxplots of the position at the inner and outer curvature, respectively; 95% confidence intervals were calculated assuming normal distribution around the intended landing zone and depicted as error bars in the graphs (SPSS 17.0, SPSS Inc., Chicago, IL, USA).

## Results

### Phantom Studies

**Fig. 3:**
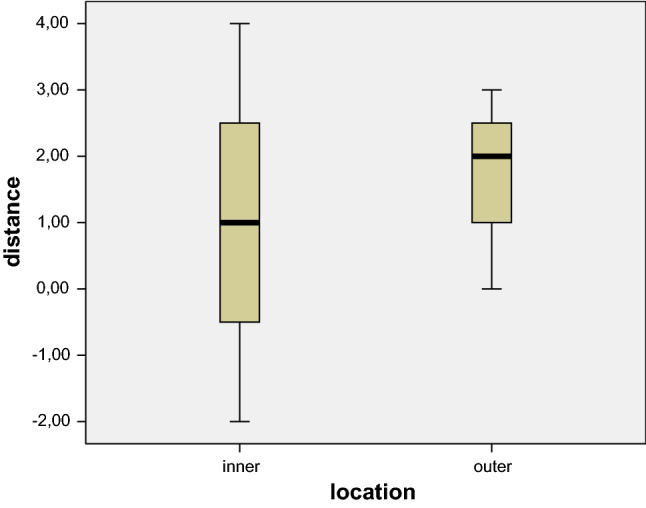
Boxplots indicating the actual location of the proximal end of the stent-graft in the phantom experiments in relation to the intended landing zone on the inner (left) and the outer (right) curvature of the aorta. The whiskers represent the actual range, the solid boxes the 2nd and 3rd quartiles, respectively

All stent-grafts were deployed with the proximal end perpendicular to the aortic axis. The results of the phantom experiments are shown in Fig. 3. At the inner curvature, the proximal end of the stent-graft was located within 2 mm proximally and 4 mm distally to the intended landing zone. At the outer curvature, the position was 0–3 mm distally to the intended landing zone. No overstenting of an arterial branch occurred.

### Cadaver Studies

The stent-grafts were successfully deployed in all four experiments. In one cadaver, the nose cone could neither be advanced through the aortic valve nor was the sinus of Valsalva deep enough to accommodate the nose piece fully (in the more recent prototypes the nosepiece was shortened). Therefore, the stent-graft had to be deployed 14 mm distally to the origin of the left coronary artery.

**Fig. 4:**
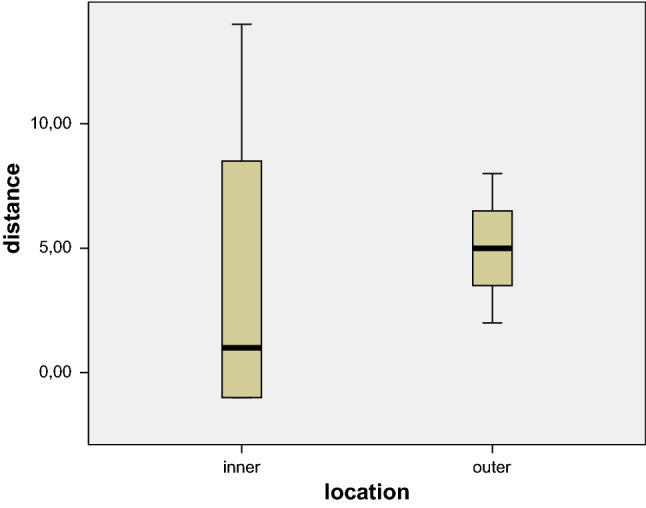
Boxplots indicating the actual location of the proximal end of the stent-graft in the cadaver experiments in relation to the intended landing zone on the inner (left) and the outer (right) curvature of the aorta. The whiskers represent the actual range, the solid boxes the 2nd and 3rd quartiles, respectively

**Fig. 5:**
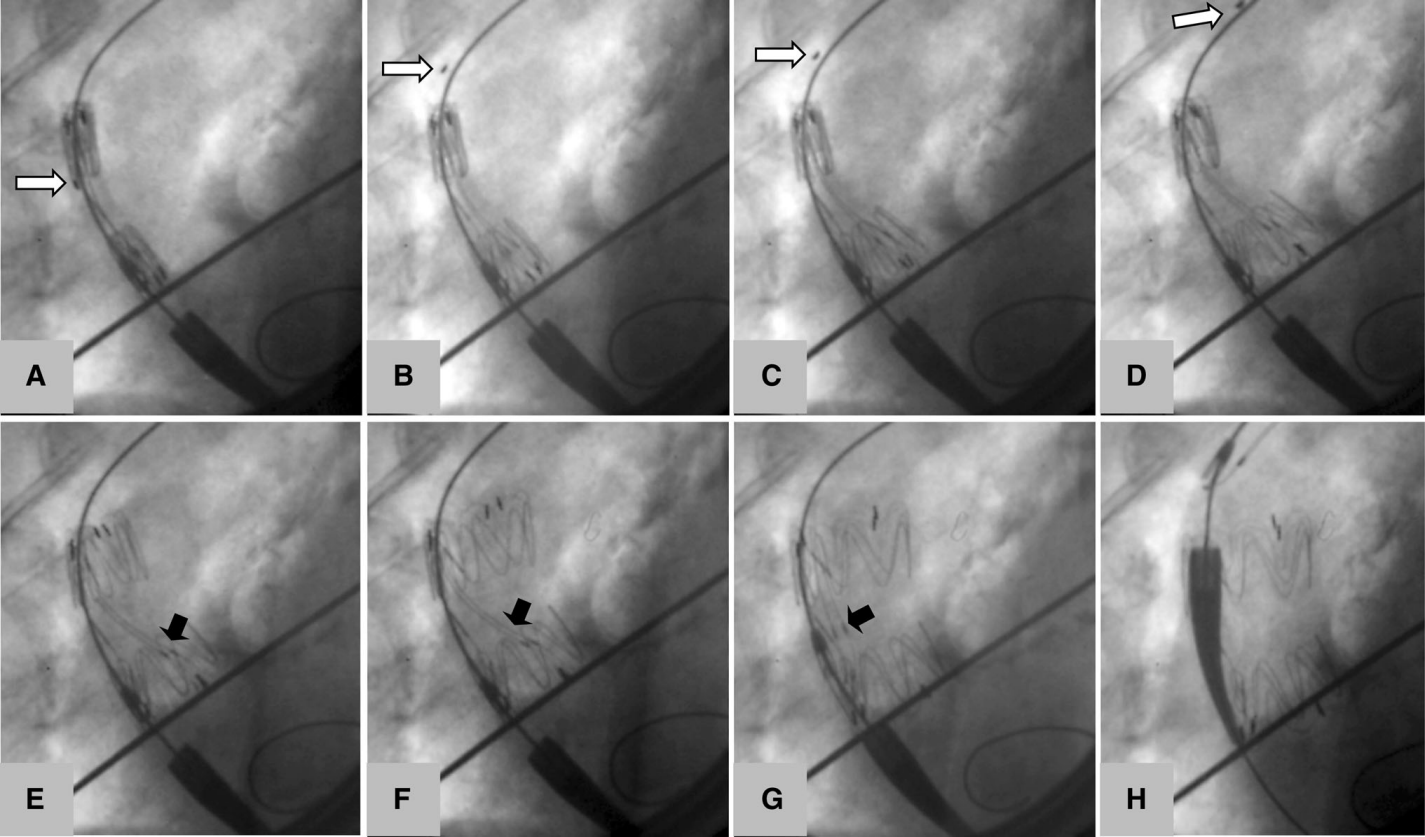
Deployment of the novel stent-graft in the ascending aorta of a human cadaver. (A) Stent-graft inside the soft 12-mm nylon sheath in deployment position (B) opening of the stent-graft by retraction of the soft sheath. Note the marker on the soft sheath gradually retracted in A–D (white arrows) (C, D). The part of the stent-graft facing the inner curvature moves controlled and perpendicular to the aortic axis toward the inner curvature of the ascending aorta (E). Note the two levers aiding in stabilizing the stent-graft during this maneuver (black arrows). (F) The stent-graft has made contact with the inner aortic curvature, the inner apices of the proximal stent segment are still held by the two levers. (G) The tip capture has been released; the two levers are now parallel to the central cannula. (H) The stent-graft is fully released; the delivery system is retracted

The distance between the actual position of the proximal stent-graft margin and the marker for the intended landing zone is shown in Fig. 4. All deployments were within 1 mm proximal and 14 mm distal to the intended landing zone on the inner curvature, and between 2 and 8 mm distal to the intended landing zone on the outer curvature (Fig. 5).

### Clinical Application

**Fig. 6:**
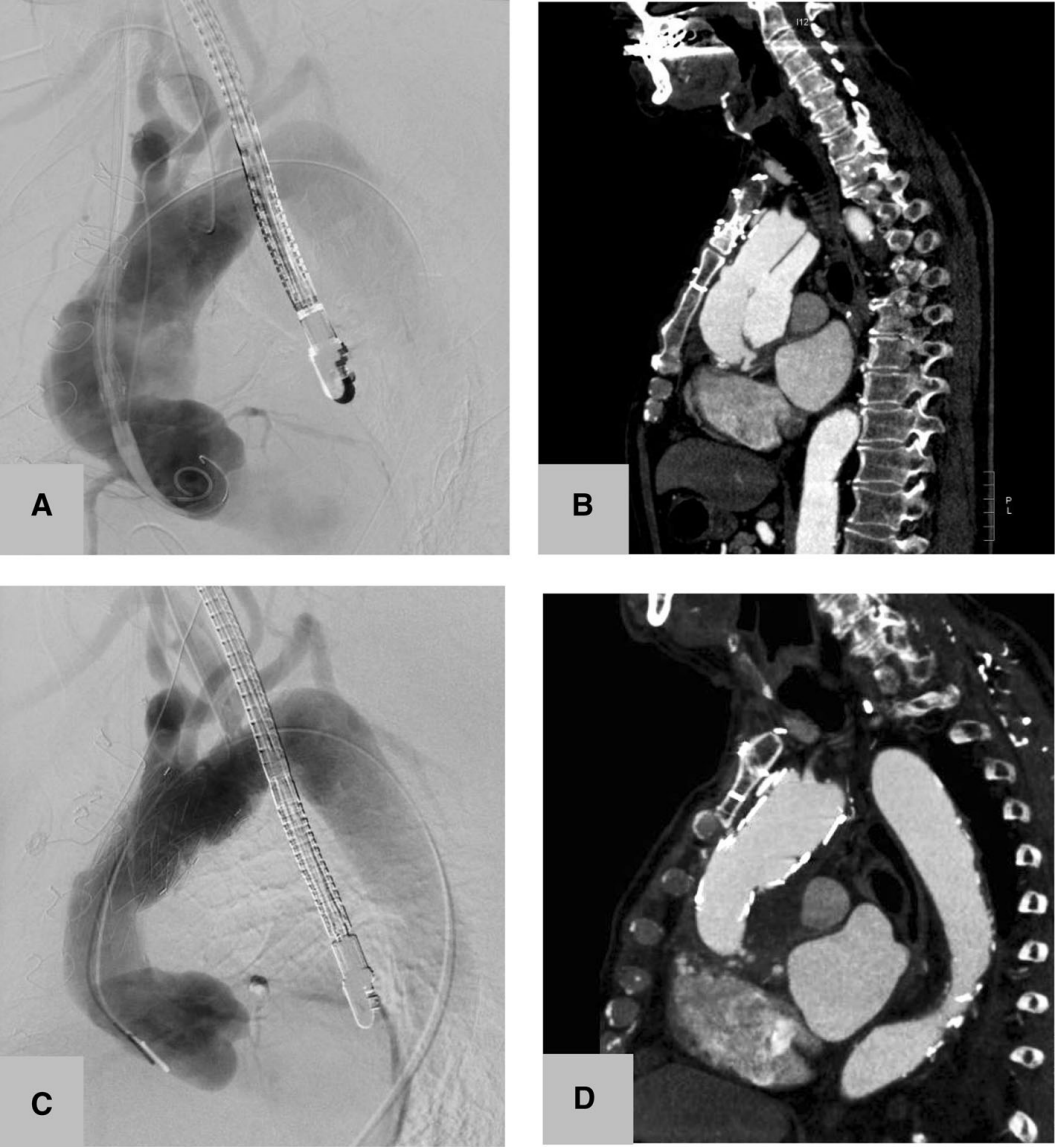
CT and digital subtraction angiography before (A, B) and after (C, D) exclusion of an ascending aortic pseudoaneurysm by deployment of 2 dedicated ascending aortic stent-grafts

Both stent-grafts were successfully deployed. The patient recovered without complications and was discharged 7 days postoperatively. The final CT scan showed a complete and sustained exclusion of the pseudoaneurysm in the arterial and in the venous phase (Fig. 6).

## Discussion

A major indication for ascending aortic stent-grafts is a pseudoaneurysm after ascending aortic replacement, if the entry tear is located at sufficient distance to the coronary and the brachiocephalic arteries [37].

Commercially available descending aortic stent-grafts are of limited use in the ascending segment due to lack of the needed diameters and lengths of their deployment systems (abdominal devices). While there exist dedicated ascending aortic stentgrafts from other manufacturers, namely Zenith Ascend from Cook, and Nexus from Endospan/Jotec as well as TAG Arise from Gore, which is in an evaluation study since 2018, the reported implantations remain sparse in the literature, and the devices are difficult to obtain. All these devices have the apices of their first stent row clasped to the proximal nose cone. From the published images as well as in our own experience, we can confirm that this deployment mode can lead to significant obliqueness of the proximal stent-graft end, with the associated risk of covering the origin of the left coronary artery (Fig. 2A, B), or stent-graft collapse.

In the ascending aortic position, the stent-graft delivery system is highly curved and exerts significant pressure to the outer curvature. The proximal end of the stent-graft on the inner and the outer curvature will end up in significantly different positions along the axis of the aorta. Using devices without tip capture mechanism, the proximal end of the stent-graft during deployment tends to migrate distally until it contacts the inner curvature. In devices with tip capture mechanisms, the tips are captured at the base of the nose cone which is located close to the aortic wall on the outer curvature. The apices of the inner part are pulled forward during opening until they contact the inner aortic wall. This can lead to an extremely oblique final position of the stent-graft (Fig. 2A, B).

Our results show that the novel ascending aortic stent-graft described in this study can be applied safely and accurately via the femoral approach. Unlike other designs, the proximal end of this ascending aortic stent-graft (with their inner apices braced by levers) facilitates alignment perpendicular to the aortic axis, resulting in a more predictable and controlled deployment (Fig. 2C, D). The pre-curved nitinol core provided the necessary rotational alignment of the stent-graft reliably.

Some basic limitations of the endovascular repair of the ascending aorta exist that cannot be overcome with a tubular stent-graft design. No matter how precisely a stent-graft may be deployed, this cannot eliminate the fact that the outcome after aortic stent-grafting highly depends on the total length of the landing zone [38]. In the ascending aorta, a landing zone is required, but many ascending aortic pathologies affect the root. In a feasibility study based on high-quality preoperative angio-CT scans, endovascular stent-grafting was retrospectively deemed feasible by the authors in 37 out of 102 patients (36%) [39]. Therefore, the majority of patients with aneurysms in the ascending aorta, including type A dissections, are currently not endovascular candidates.

The implantation of stent-grafts in the ascending aorta in the majority of reported cases was effective [6–31]. Most patients had subacute type A dissections or were diagnosed with pseudoaneurysms at variable time intervals after open surgical procedures. Eleven series of 2–15 patients were described in the literature [9, 10, 13, 16, 22, 23, 26, 28–31]. While mostly thoracic stent-grafts are being used [40], physician-modifications to off-the-shelf devices [6, 10, 12] or custom-made stent-grafts may be more suitable to overcome these limitations [16, 25–27, 31].

Limitations of this study include the lack of different pressure and perfusion situations. However, given the option to deploy the stent-graft under very low flow using, e.g., rapid pacing or temporary cardiac arrest, our first investigations addressed stationary conditions. We did not test at different stent-graft and aortic diameters, and the case numbers are low. Potentially, placement becomes more erratic in larger diameters. Ongoing multi-center data evaluations will further address clinical outcomes and deployment accuracies. So far, no long-term results (>3 years) are available. Moreover, the long-term hemodynamic consequences are currently unknown. Ascending aortic stent-grafting is a feasible, but experimental treatment which comes into consideration only for selected patients and preconditions at the site of the aortic lesion.

## Conclusion

A novel stent-graft designed specifically for the ascending aorta has been successfully tested in phantoms, cadavers, and clinically. The stent-graft expands reliably perpendicular to the aortic axis and can be deployed with good accuracy. As with all stent-graft designs, a sufficient landing zone, both proximal and distal, with enough leeway to the coronary arteries and the brachiocephalic trunk is a prerequisite.
